# Prevention and health promotion from theory to practice: The interprofessional MeMPE Summer University for students of Medicine, Master of Public Health and Epidemiology

**DOI:** 10.3205/zma001071

**Published:** 2016-11-15

**Authors:** Nadja Idler, Johanna Huber, Sabine von Mutius, Lena Welbergen, Martin R. Fischer

**Affiliations:** 1 LMU Munich, Medical Faculty, Support Program Lehre@LMU, Munich, Germany; 2 University Hospital of LMU Munich, Institut für Didaktik und Ausbildungsforschung in der Medizin, Munich, Germany; 3 LMU Munich, Medical Faculty, Institute for Information Processing, Biometry and Epidemiology, Munich, Germany; 4 University Hospital of LMU Munich, Dr. von Haunersches Children's Hospital, Munich, Germany; 5 University Hospital of LMU Munich, Institut für Didaktik und Ausbildungsforschung in der Medizin, Munich, Germany

**Keywords:** medical education, interprofessional education, prevention, health promotion, public health, epidemiology

## Abstract

**Objective: **During the 2015 summer semester of Munich’s Ludwig Maximilian University (LMU) medical school, the pilot project “MeMPE Summer University – An Interprofessional Seminar on Prevention and Health Promotion” was implemented as a compulsory elective subject. In 90 teaching units of 45 minutes each, 20 students from the degree programs of Medicine, Master of Public Health and Master of Science Epidemiology (MeMPE) completed modules in theoretical introduction, scientific project work as well as practical assignments and conference attendance.

**Methods: **The project was evaluated by students using pre- and post-project questionnaires (26 and 57 items, evaluated on a Five-level Likert scale of 1=“fully agree” to 5=“fully disagree”). The evaluation interviews of the instruction participants were recorded, transcribed and analyzed according to Mayring’s qualitative content analysis.

**Results: **Questionnaire response rate was 100 %. In pre/post comparison, the students reported an improvement in factual knowledge (pre median=3.0; post median=2.0; p<0.0001), in scientific work (pre median=3.0; post median=1.0; p<0.0001) and in interprofessional work (pre median=2.0; post median=1.0; p=0.024). In 18 interviews, the instructors largely expressed their motivation to participate in the project again.

**Conclusion:** The MeMPE Summer University can serve as an example of best practice for interprofessional communication of prevention and health-promotion topics in theory and practice. The evaluation results show that the project enjoyed a high level of acceptance among students and instructors, and that it should be conducted in a revised version again in 2016.

## Zusammenfassung

**Zielsetzung: **An der Medizinischen Fakultät der LMU München wurde das Wahlpflichtfach „MeMPE Summer University – Ein interprofessionelles Seminar zu Prävention und Gesundheitsförderung“ im Sommersemester 2015 als Pilotprojekt umgesetzt. 20 Studierende der Studiengänge Medizin, Master of Public Health und Master of Science Epidemiology (MeMPE) haben in 90 Unterrichtseinheiten je 45 Minuten ein Modul mit theoretischer Einführung, wissenschaftlicher Projektarbeit sowie ein Modul mit Praxiseinsatz und Kongressbesuch absolviert.

**Methodik: **Das Projekt wurde bei den Studierenden mittels Prä- und Post-Fragebogen evaluiert (26 und 57 Items, Bewertung mittels fünfstufiger Likert-Skala von 1=„stimme voll zu“ bis 5=„stimme gar nicht zu“). Die Evaluationsinterviews der Lehrbeteiligten wurden aufgezeichnet, transkribiert und mit der qualitativen Inhaltsanalyse nach Mayring ausgewertet.

**Ergebnisse: **Die Fragebogen-Rücklaufquote betrug 100%. Die Studierenden berichten im Prä-/Post-Vergleich eine Verbesserung des Faktenwissens (Prä-Median=3,0; Post-Median=2,0; p<0,0001), des wissenschaftlichen Arbeitens (Prä-Median=3,0; Post-Median=1,0; p<0,0001) und des interprofessionellen Arbeitens (Prä-Median=2,0; Post-Median=1,0; p=0,024). In 18 Interviews äußern die Lehrenden überwiegend ihre Motivation zur erneuten Projektteilnahme.

**Schlussfolgerung: **Die MeMPE-Summer-University kann als Best-Practice-Beispiel für die interprofessionelle Vermittlung von Präventions- und Gesundheitsförderungsthemen in Theorie und Praxis dienen. Die Evaluationsergebnisse zeigen, dass das Pilotprojekt eine hohe Akzeptanz bei Studierenden und Lehrenden erfahren hat und in überarbeiteter Form im Jahr 2016 erneut durchgeführt werden soll.

## Introduction

Since the ninth revision of the German Medical Licensure Regulation (ÄAppO), “prevention and health promotion” have defined one of 14 interdisciplinary fields in the second phase of medical education which require proof of achievement and are given grades [http://www.gesetze-im-internet.de/_appro_2002/BJNR240500002.html, cited May, 1, 2016], [1]. The implementation of the interdisciplinary field “prevention and health promotion” shows differences between the medical faculties in regard to coordinative and content responsibility, time scope, the inclusion of healthcare practice as well as the definition of the proof of achievement [1].

The medical faculty of the Ludwig Maximilian University of Munich (LMU) is implementing the interdiscipline Prevention and Health Promotion for students of human medicine in the framework of the so-called longitudinal course, with three 90-minute lectures and two 90-minute seminars. The longitudinal course takes place throughout the entire degree program, with the intention of familiarizing students with “the physician’s role and responsibility with patients and in society” [https://www.mecum-online.de/de/studium/longitudinalkurs/index.html, cited May, 1, 2016]. For students in the masters programs Public Health and Epidemiology, the medical faculty offers the compulsory elective “Prevention and Health Promotion” (12 ECTS).

To date, this course has not included healthcare practice. Available since October 2014, the government-funded program “Lehre@LMU” offers students practice-oriented projects in order to intensify student participation. The intention here is to address areas which are not covered in the Medical Curriculum Munich (MeCuM) or in the curricula of the masters programs, for example primary care in rural areas and public-health practice assignments [http://www.uni-muenchen.de/studium/lehre_at_lmu/index.html, cited May 1, 2016]. The goal of the present project report is to describe a Summer-University model in accordance with best practice that facilitates the conveyance of prevention and health-promotion topics in theory and practice. Furthermore, methods and results of project evaluation shall be reported. 

## Project description

During the summer semester of 2015, the LMU medical faculty’s Lehre@LMU support program implemented the “MeMPE Summer University – An Interprofessional Seminar on Prevention and Health Promotion” from September 14 to September 25, 2015 as a pilot project and compulsory elective (in accordance with the ÄAppO). The abbreviation MeMPE describes the interprofessional character of the seminar and stands for “Medicine, Master of Public Health and Master of Science Epidemiology” (degree programs in human medicine as well as master’s programs in public health and epidemiology). The students work together in interprofessional tandems consisting of one student of human medicine and one student from a master’s program. Because the quality of future cooperation between healthcare practice and research also relies on the degree of interprofessionality integrated in the educational system [2], [3], the seminar is meant to afford students an early exchange with content-related disciplines so that the modes of thought and work methods of future cooperation partners can be familiarized and reflected upon at an early stage.

Until now, these degree programs have had no joint courses at the LMU Munich. To our knowledge, no comparable teaching concept exists in German-speaking Europe.

The two-week seminar consists of 90 teaching units (TU) of 45 minutes each that are completed on ten course days. The seminar is divided into one theoretical (T) and one practical (P) module with the submodules T1 “Theoretical Introduction” (12 TU), T2 “Scientific Project Work” (28 TU), P1 “Practical Assignment” (19 TU) and P2 “Conference Attendance” (31 TU).

### Learning objectives and program schedule

A1 (see attachment 1 ) shows the learning objectives of the MeMPE Summer University. Chronologically, the seminar was held as follows (see also seminar schedule, A2 attachment 2 ): The submodule T1 “Theoretical Introduction” (days one and two) served the purpose of introducing the students to the subject matter through lectures as well as preparing them for the mapping out of their scientific project.

On the third and fourth days (submodule P1 “Practical Assignment”), the students completed an assignment in their selected focus area of either rural healthcare, public health department or project risk assessment (“Risikolotse”). The project Risikolotse.de from the Helmholtz Zentrum München is dedicated to the calculation and communication of individual risks of breast cancer. In their focus areas, the students were involved in projects of prevention or health promotion in order to identify subject areas for the preparation of their scientific project.

Submodule T2, “Scientific Project Work” (days five, six, seven) consisted of a five-page, structured summary and a structured 11- to 13-minute presentation which was prepared in tandem according to specifications of a learning-objectives log book. Each tandem group had the opportunity to discuss and elaborate the project idea with a mentor in two TUs. The mentors were experienced lecturers and practitioners from the field of prevention and health promotion.

In the framework of submodule P2, conference attendance was arranged in collaboration with the conference “Daten gewinnen, Wissen nutzen für die Praxis von Prävention und Versorgung” (“Collecting Data, Implementing Knowledge in the Practice of Prevention and Healthcare”) from September 23 to September 25, 2015 in Regensburg, Germany. In seven TUs, attendance was required (peer-to-peer short presentations and final short presentations with a public audience). The remaining time was free for attending academic talks at the students’ discretion. 

#### Implementation and participants

Twenty students successfully completed the MeMPE Summer University: ten students of human medicine, eight students of public health and two students of epidemiology. Six interprofessional tandems or tridems were formed. 

Project cooperation partners were the Landesamt für Gesundheit und Lebensmittelsicherheit (LGL – Bavarian State Office for Health and Food Safety), the Pettenkofer School of Public Health (PSPH) and the Bayerische Landesärztekammer (BLÄK – Bavarian State Chamber of Physicians). The participation of four public health offices, of the project Risikolotse.de as well as that of three rural medical practices made the students’ practical assignments possible. In the framework of this project, rural medical practice indicates a general medical practice in a rural setting, i.e., outside of a metropolitan area. 

## Evaluation methods

The Summer University was evaluated by means of questionnaires (pre and post survey) by the students and through short, structured interviews with the participating lecturers, mentors and practice supervisors.

### Survey questionnaires

The survey took place on the first day, before the seminar commenced (pre questionnaire), and on the last day, after the seminar had ended (post questionnaire), using machine-readable forms.

The pre questionnaire consisted of 26 items for self-evaluation of learning objectives using a five-level Likert scale of 1=“fully agree” to 5=“fully disagree”. The participants were also able to choose the option “not specified (n.s.)”. Of the 26 items, 25 were assigned to the following subscale based on their thematic affiliation:

1. Factual knowledge on the subjects of prevention and health promotion (15 items),
2. scientific work (7 items),
3. interprofessional work (3 items).

One item addressed prior knowledge on the subjects of prevention and health promotion. 

The post questionnaire consisted of 57 items, of which 56 could be rated on a five-level Likert scale with the supplementary optional answer “n.s.”. As in the pre questionnaire, 26 items covered the self-evaluation of learning objectives for a pre/post comparison of the mentioned subscales. Furthermore, the post questionnaire covered four further subscales in order to capture aspects of the instructional program and of the practical assignment:

1. Organization (5 items),
2. supervision (3 items),
3. didactics (11 items) and
4. overall assessment of learning results (11 items).

Another item, with the response categories of A to H, covered the students’ motivation for taking part in the Summer University. 

The questionnaires were created and evaluated using Zensus direkt (version 5.2.0p4) software. The questionnaires were scanned for automated evaluation. Each questionnaire was examined for correct data entry. Zensus direkt was used to calculate the absolute and relative frequency, the median and the range for each item. 

Each subscale’s median was calculated with the statistical analysis program SPSS. Subscale median was calculated as follows: Firstly, a median was calculated for each participant from items belonging to a specific subscale. Subsequently, a pooled median was calculated within each subscale from the individual medians of the participants. The median values comprised decimal places (five-level Likert scale: 1.0=“fully agree” to 5.0=“fully disagree”). The Mann-Whitney U test for independent samples in nonparametric data with a significance level of 0.05 was chosen for the pre/post comparison of learning objectives in the three subscales “factual knowledge”, “scientific work” and “interprofessional work”. 

#### Short, structured interviews

The short, structured interviews were conducted using an interview guide. Practice supervisors, lecturers and mentors were asked questions on the following topics:

1. Motivation for collaboration,
2. feasibility of further collaboration,
3. usefulness of the support by the project organization Lehre@LMU during project preparation and implementation,
4. possibilities for the improvement of support.

Additionally, the mentors and practice supervisors were asked in three further questions to assess the students’ previous knowledge and level of competence. Lecturers were asked questions on the students’ previous knowledge. 

All of the interviewees were provided with data protection information and gave declarations of consent. The approximately ten-minute interviews were conducted by telephone, and audio files of the sessions were created with field recorders. The audio interviews were transcribed verbatim. The evaluation of the interviews took place in qualitative content analysis according to Mayring [4]. The basic technique used to establish the core statements was a summarizing content analysis [4] in multiple steps:

1. By means of the interview question, a coding scheme was developed by creating main categories and, as necessary, subcodes* (example: For the question addressing willingness to participate future implementations, the main category “desire for future participation” as well as the subcodes “yes/no”, “reason” and “comment” were created)*,
2. the relevant statements from the transcription text were extracted,
3. paraphrased,
4. generalized and
5. allocated within the coding scheme.

Steps 3 and 4 could be performed repeatedly until a level of abstraction was reached that allowed the statements’ allocation within the coding scheme. The results were summarized and presented in bar graphs using the software Microsoft Excel (version 2013).

## Evaluation results

### Results of the survey with questionnaires

Questionnaire-return rate was 100% (N=21 pre, and N=20 post questionnaires). The presence of a duplicate among the pre questionnaires was suspected but could not be identified, therefore, all pre questionnaires are included in the evaluation. Response refusals were recorded a total of ten times in the pre questionnaire and 23 times in the post questionnaire.

**Figure 1:**
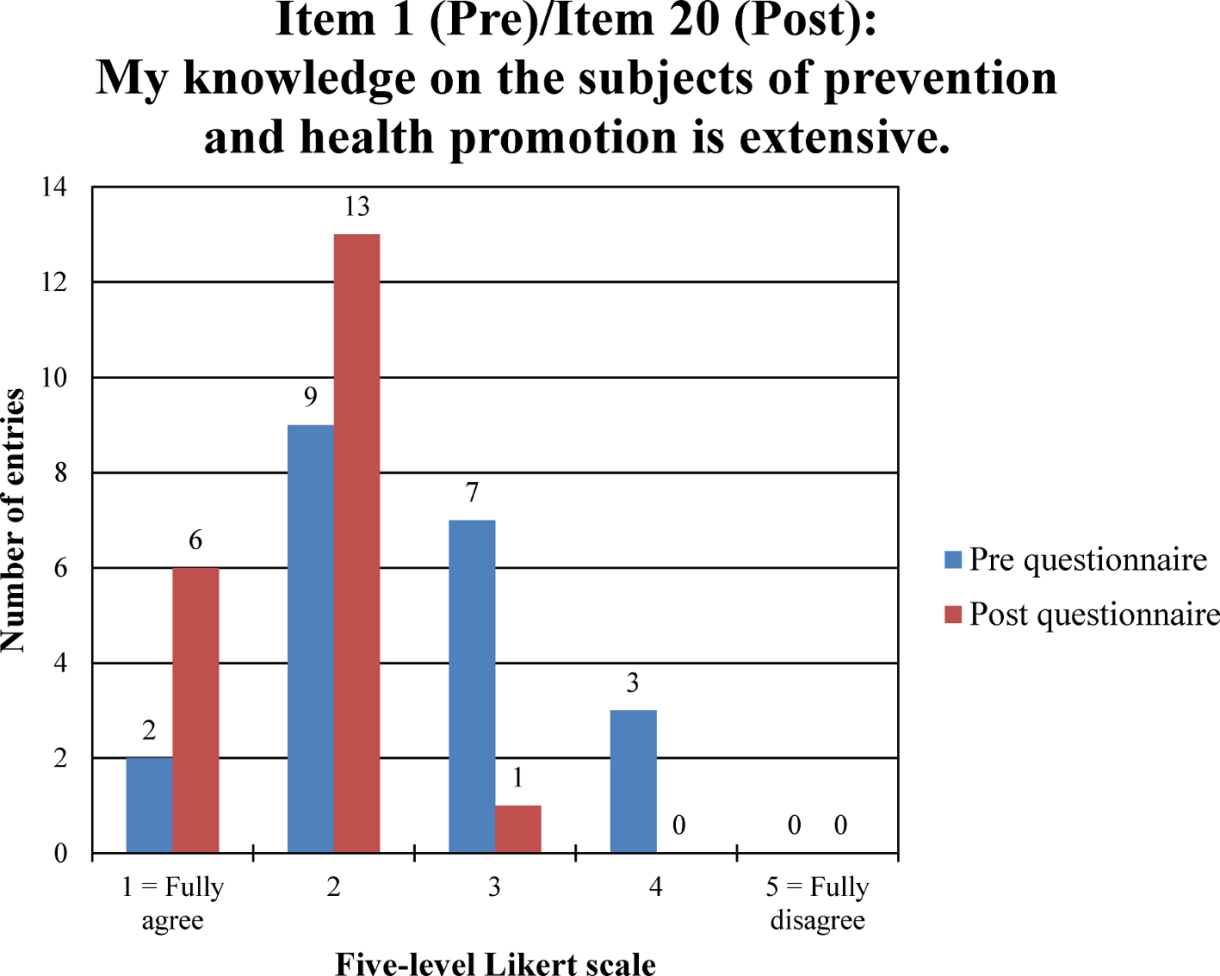
Frequency distribution “(prior) knowledge on the subjects of prevention and health promotion” in pre/post comparison

According to the pre questionnaire, participants’ knowledge (item 1) on the subjects of prevention and health promotion prior to the start of the seminar is high (median=2.0; range=3.0). There is no shift in the median value in the post questionnaire (item 20), but the range is decreased (median=2.0; range=2.0) (see figure 1 (Fig. 1)). 

Table 1 (Tab. 1) shows the results of the three subscales on the students’ self-evaluation. In pre/post comparison, the change in median values is highly significant (factual knowledge, p<0.0001; scientific work, p<0.0001) or significant (interprofessional work, p=0.024). The students attest to a considerable improvement in their factual knowledge as well as their competencies in respect to scientific work and interprofessional work. 

Table 2 (Tab. 2) shows the results of the four additional subscales from the post questionnaire.

The students were satisfied with the organization and didactic concept of the seminar (median=2.0; range=2.0; respectively median=2.0; range=3.0) and very satisfied with the supervision of the practical assignments and the supervision during the entire seminar (median=1.0; range=3.0). The overall assessment of learning results was also good (median=2.0; range=2.0). The reasons given for participation motivation were primarily interest in the topic (N=16), enhancing knowledge (N=10) as well as familiarization with the professional fields (N=20).

The complete results from all of the items (median and range values) entered in the subscales are reported in A3 (see attachment 3 ).

#### Results of the short, structured interviews

**Figure 2:**
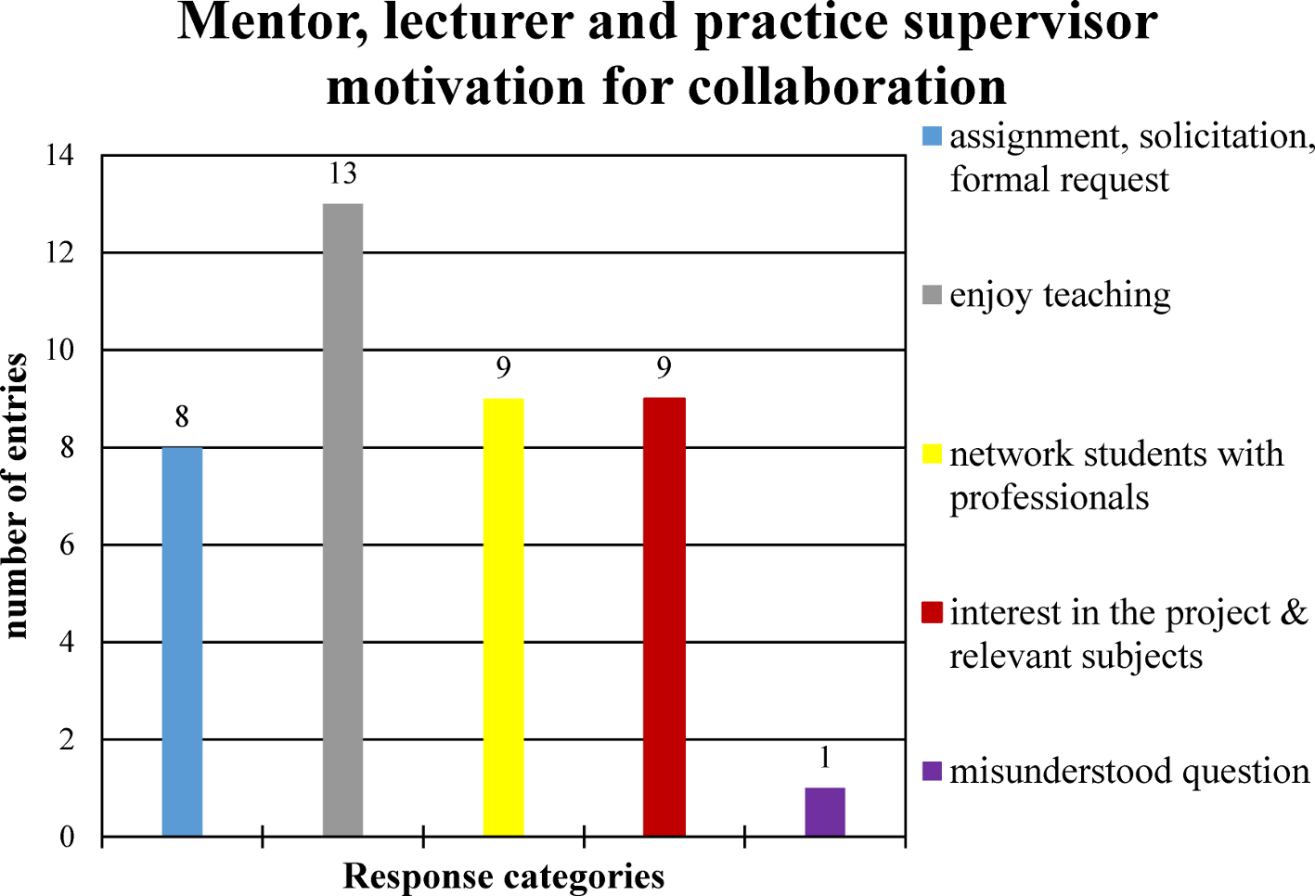
Mentor, lecturer and practice supervisor motivation for collaboration

A total of 18 short, structured interviews were conducted (N=5 mentors, N=10 lecturers and N=8 practice supervisors; some overlapping). In the subject area “motivation for collaboration”, 40 answers were identified for which the content could be assigned to five categories (see Figure 2 (Fig. 2)).

**Figure 3:**
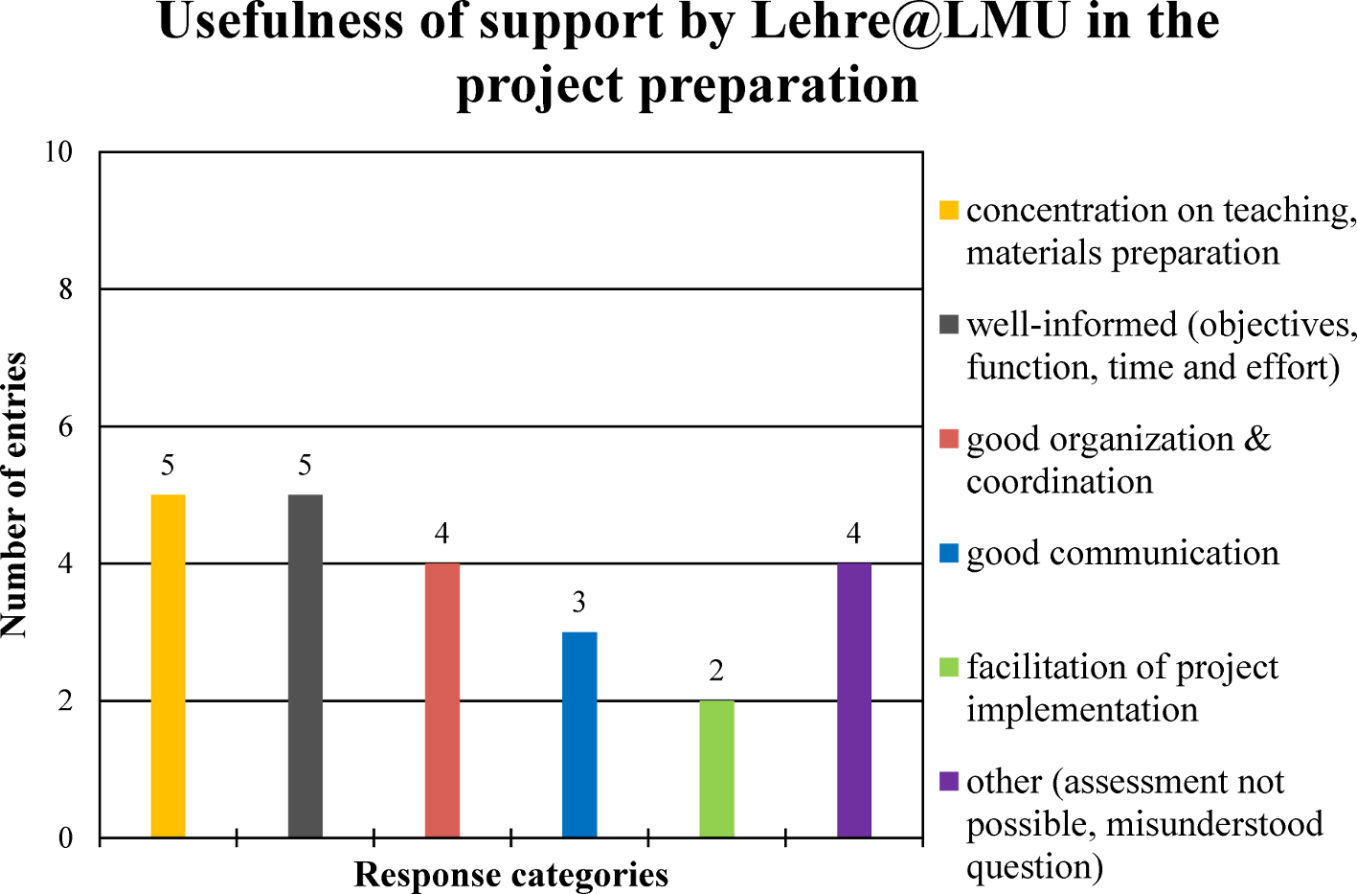
Usefulness of support by Lehre@LMU in project preparation

**Figure 4:**
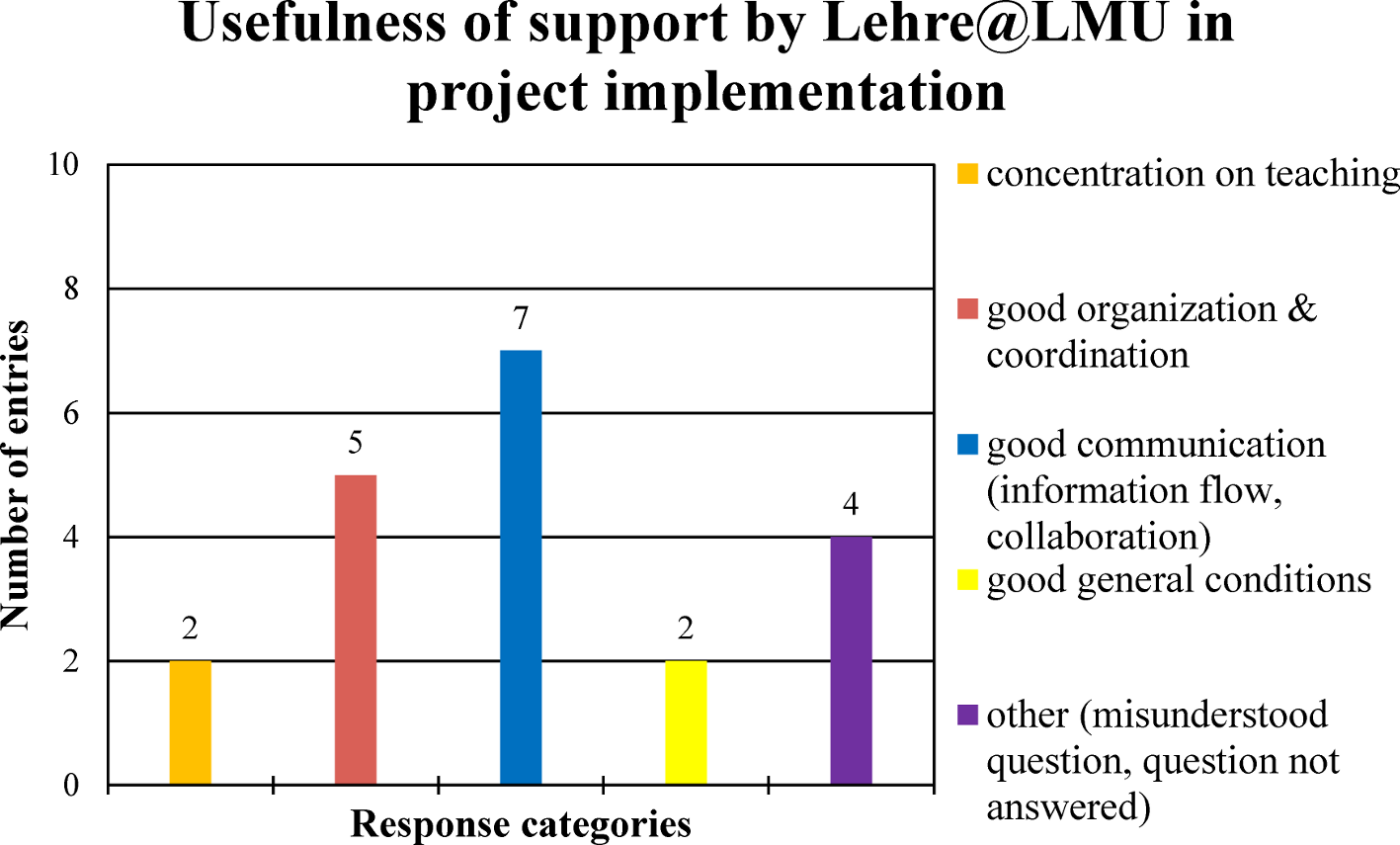
Usefulness of support by Lehre@LMU in project implementation

In response to the question of “usefulness of the support by the project organization Lehre@LMU during project preparation and during project implementation”, the interviewees made 23 and 20 entries respectively (see Figure 3 (Fig. 3) and Figure 4 (Fig. 4)).

In order to improve support performance, three mentors call for an “optimization of supervision ratios in mentoring”. Lecturers ask for a “teaching introduction for seminar design” (N=1) and for improvement of “lecturer visibility” (N=1). All of the mentors and lecturers interviewed would take part in the project again. Five of the practice supervisors agree to future collaboration. The reasons given were “pleasure of collaboration with students” (N=2) and “assistance for students in their professional pursuits” (N=5). 

Three practice supervisors observe “little” previous knowledge of the general content of practical assignments among the students. Four practice supervisors, however, attest to students’ specific “medical knowledge from clinics and general physician practices”. Practice supervisors emphasize students’ exceptional competencies in “critical and differentiated working methods” (N=2), “interest” (N=3) as well as “communication strength and collegiality” (N=4). It is difficult for the mentors to estimate the students’ previous knowledge (N=3). They attest to the students’ competencies in “research interest and basic inquisitiveness” (N=5), in “independent work methods” (N=2) and in “scientific work methods” (N=4). According to the lecturers (N=4), the students displayed general prior knowledge, but the assessment of specific prior knowledge proved difficult (N=3). 

## Discussion

The first implementation of the interprofessional MeMPE Summer University as a pilot project was successful. In the pre/post comparison, the students noted a marked increase in learning through seminar participation. The participating instructors also expressed high satisfaction levels and motivation for future project collaboration. 

In accordance with the recommendations of the German Association for Medical Education’s (GMA) committee “Interprofessional Education in Health Professions”, the principle of interprofessionality was integrated into the seminar in order to prepare the students for interprofessional collaboration in their future careers [3]. In 2011, the Interprofessional Education Collaborative Expert Panel developed four competency domains for interprofessional collaborative practice in the United States [Retrieved May 1, 2016 from http://www.aacn.nche.edu/education-resources/ipecreport.pdf]. In particular, the content of the competency domains “interprofessional communication” and “teamwork” are incorporated in the MeMPE Summer University [Retrieved May 1, 2016 from http://www.aacn.nche.edu/education-resources/ipecreport.pdf]. Furthermore, the didactic concepts of peer teaching and mentoring are integrated in the project [5], [6], [7].

A limitation of the project can be seen in its implementation on a single site at the moment and in the low number of participants (N=20). The number of students participating in the summer semester of 2016 is to be augmented through early promotion of the seminar. The creation of exclusively interprofessional tandems is also being targeted. According to the results of the post-evaluation, several students would like to meet the entire group as well as their tandem partners at an earlier stage. A pre-seminar meeting is being considered, during which the students can choose their tandem partners for the practical project themselves. 

Motivation for the future collaboration of the participating rural medical practices, public health offices and the project “Risikolotse” is also being targeted. The integration of further cooperation partners, such as the project “Health Promotion in General Practice – Obstacles and Opportunities” from the LMU medical faculty in cooperation with the Integrative Health Promotion studies program of the HAW Coburg, is being planned. 

Furthermore, the reduction of the P2 module “Conference Attendance” to a one-day lecture event by the medical faculty of the LMU Munich is being considered for the benefit of modules T2 and P1. Several students expressed a desire for more time for the preparation of their scientific project (T2), for discussions regarding project design and for practical assignments (P1). In order to optimize the content of the module T1, a closer match with the learning content of the medical study program as well as with the curricula of the study programs Master of Public Health and Master of Science Epidemiology is being striven for. In the long term, a student survey comparable to that of Klement et al. at the Martin Luther University Halle-Wittenberg medical faculty could be conducted [8]. Said survey polled medical students on their preferences, stances and previous knowledge in regard to the subject areas of prevention and health promotion in order to determine requirements concerning communication of instructional content [8]. 

In order to properly document the achievement of learning objectives at an individual level, the questionnaire evaluation (pre and post) should be individually attributable in future implementation. To this end, individual codes should be noted on the questionnaires.

Overall, on the basis of the evaluation data, the MeMPE Summer University can be regarded as a best-practice model for conveying the theory and practice of prevention and health promotion. 

Lehre@LMU recommends its implementation in other medical faculties at universities in Germany as well. 

## Acknowledgements

The realization of the project was made possible by the financial support of the Lehre@LMU funding program (“Qualitätspakt Lehre from the German Federal Ministry of Education and Research (BMBF)) for the promotion of practice orientation. 

Thanks goes to all of the lecturers, mentors and project coordinators participating in the MeMPE Summer University. Special thanks goes to the staff of the LGL. 

We thank the practices of Dr. med. Michael Rosenberger, Dr. med. Günter Oberprieler and Dr. med. Wolfgang Blank, the public health authorities of Regensburg, Erlangen, Erding and Weilheim-Schongau as well as the project Risikolotse of the Helmholtz Zentrum München for making the students’ practical assignments possible. 

We thank the Bavarian State Chamber of Physicians for their non-material support and announcement of the seminar, as well as for the opportunity of presenting the Summer University as an innovative project in university medical education at this year’s 74th Bavarian Medical Assembly in Deggendorf. 

## Index of abbreviations

- ÄAppO: Ärztliche Approbationsordnung (German Medical Licensure Regulation)
- BLÄK: Bayerische Landesärztekammer (Bavarian State Chamber of Physicians)
- BMBF: Bundesministerium für Bildung und Forschung (German Federal Ministry of Education and Research)
- ECTS: European Credit Transfer System
- GMA: Gesellschaft für Medizinische Ausbildung (German Association for Medical Education)
- HAW: Hochschule für angewandte Wissenschaften (University of Applied Sciences)
- n.s.: not specified
- LGL: Landesamt für Gesundheit und Lebensmittelsicherheit (Bavarian State Office for Health and Food Safety)
- LMU: Ludwig-Maximilians-Universität (Ludwig Maximilian University)
- MeCuM: Medizinisches Curriculum München (Medical Curriculum Munich)
- MeMPE: Medicine, Master of Public Health und Master of Science Epidemiology
- PSPH: Pettenkofer School of Public Health
- TU: teaching unit

## Competing interests

The authors declare that they have no competing interests.

## Supplementary Material

A1: Modules, time scope and learning objectives of the MeMPE Summer University

A2: MeMPE Summer University 2015 Schedule

A3: Subscales of the pre and post questionnaires, with allocation of items, median values and range values

## Figures and Tables

**Table 1:**
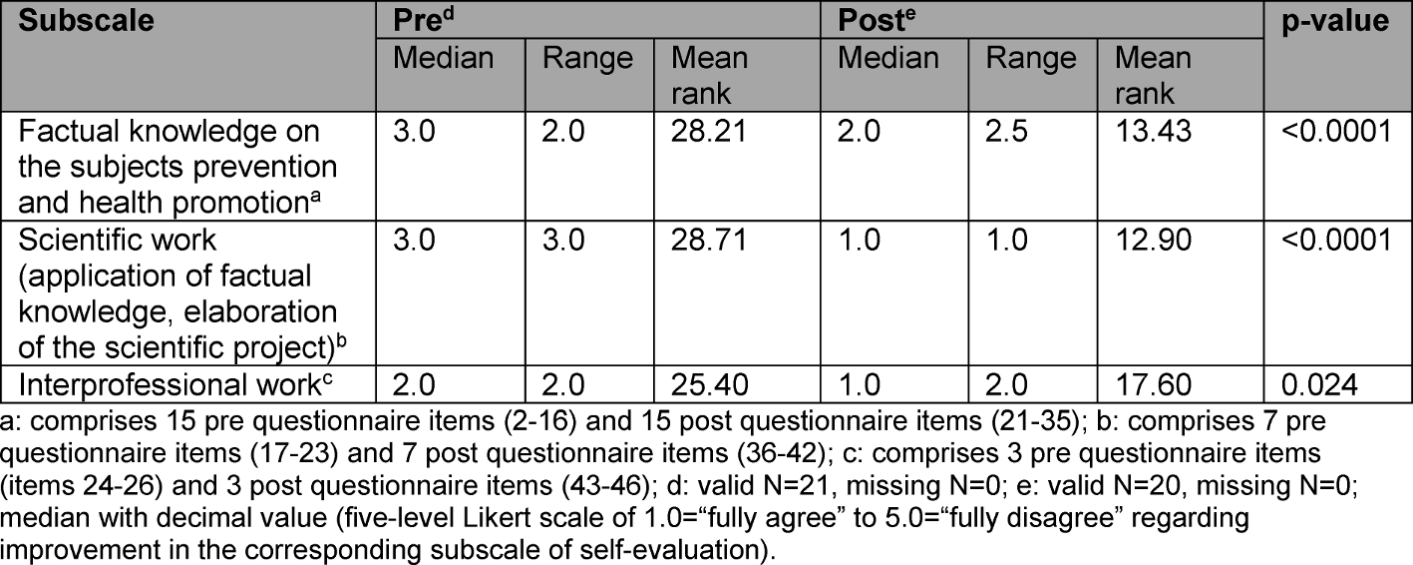
Subscales in self-evaluation of learning objectives in pre/post comparison

**Table 2:**
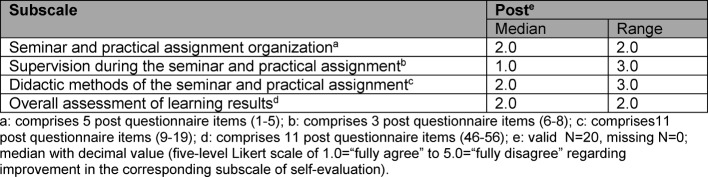
Subscales for the post survey on organization, supervision, teaching methods and overall assessment
