# Supplementary material for: Prevention and health promotion from theory to practice: The interprofessional MeMPE Summer University for students of Medicine, Master of Public Health and Epidemiology
Source: GMS J Med Educ. 2016 Nov 15;33(5):Doc72. doi: 10.3205/zma001071 (PMC5135415; doi:10.3205/zma001071)
Supplement: A1: Modules, time scope and learning objectives of the MeMPE Summer University [file JME-33-72-s-001.pdf]

A1: Modules, time scope and learning objectives of the MeMPE Summer University

Module	Number of TUs	Day*	Learning objectives: Upon completion of the module, students should be able to..
**Theory Module (T)**			
T1: theory introduction	12 required	1, 2	• name the contents of the Ottawa Charter for Health Promotion;<br>• describe the Model of Salutogenesis;<br>• name the fields of action and guiding principles of health promotion;<br>• outline the (community-) setting approach;<br>• discern target groups and points of time for intervention in the realm of prevention;<br>• describe chains of events in prevention and desired outcomes in health promotion;<br>• outline direct and indirect means of conveying public prevention messages;<br>• describe different media uses as a basis for conveying public prevention messages;<br>• name different forms of approach via mass communication and personnel communication;<br>• name the fundamental principles of physician-patient communication;<br>• name the core contents of communicating prevention information in physician-patient direct consultation;<br>• describe strategies of communicating prevention information in physician-patient direct consultation;<br>• name the data sources of the Information System of Health Monitoring (Federal and Bavarian);<br>• use the data sources of the Information System of Health Monitoring (Federal and Bavarian) for their project work;<br>• name the steps in project planning;<br>• use the steps of project planning to design their project;<br>• name the criteria of Good Practice;<br>• use the criteria of Good Practice in designing their project.
T2: scientific project work**	28 required (of which 2 men-toring)	5, 6, 7	• formulate a well-founded scientific problem statement grounded in the area of prevention and health promotion;<br>• elaborate a scientific project report according to specific guidelines;<br>• elaborate a scientific project presentation according to specific guidelines.
**Practical Module (P)**			
P1: practical assignment**	19 required	3, 4	*Practical assignment in a rural medical practice:*<br>• outline the practical implementation of prevention and health promotion in a rural medical practice;<br>• describe the physician's function in a general practice in a rural setting;<br>• identify an appropriate topic area in prevention and health promotion in preparation for their scientific project work.

Module	Number of TUs	Day*	Learning objectives: Upon completion of the module, students should be able to..
			Practical assignment in a public health office:<br>• outline work on a prevention project in a public health office;<br>• identify an appropriate topic area in prevention and health promotion in preparation for their scientific project work.<br><br>*Project Risikolotse practical assignment – dependent on selected workshop option:*<br>*Workshop Option 1:*<br>• outline the current state of screening in Germany.<br>*Workshop Option 2:*<br>• name the risk assessment models IBIS; BOADICEA and GAIL;<br>• calculate risks using the corresponding risk-assessment software.<br>*Workshop Option 3:*<br>• name the fundamental principles of risk communication;<br>• communicate breast-cancer and screening risks.<br>*Independent of selected workshop option:*<br>• identify an appropriate topic area in prevention and health promotion in preparation for their scientific project work.
P2: conference attendance	31 (of which 7 required**)	8, 9, 10	By presenting their own projects, the students should improve their skills in public presentation.
**Learning objectives for all modules**	n.s.	in the course of the seminar	• conduct interprofessional exchange with a tandem partner;<br>• elaborate a project with a tandem partner;<br>• conduct interprofessional exchange with peers (entire group of students).

Abbreviations: P: practical module; T: theoretical module; TU: teaching unit (45 minutes each)

*Seminar day on which the module takes place; **The students complete this module in an interprofessional tandem.
