# Supplementary material for: Prevention and health promotion from theory to practice: The interprofessional MeMPE Summer University for students of Medicine, Master of Public Health and Epidemiology
Source: GMS J Med Educ. 2016 Nov 15;33(5):Doc72. doi: 10.3205/zma001071 (PMC5135415; doi:10.3205/zma001071)
Supplement: A2: MeMPE Summer University 2015 Schedule [file JME-33-72-s-002.pdf]

A2: MeMPE Summer University 2015 Schedule

	**Monday, September 14 (Auditorium II, Großhadern)**	**Tuesday, September 15 (Auditorium II, Großhadern)**	**Wednesday, September 16**	**Thursday, September 17**	**Friday, September 18 (Auditorium II, Großhadern)**
**09:00**		Presentation of three subject areas, focus areas prevention and health promotion<br>• Rural medical practice *Idler*<br>• ÖGD (Public Health Service) *Socher*<br>• Project Risikolotse *Quante/Strahwald*	Practical assignment in the respective focus area<br><br>Individual site and time according to tandem – to be announced individually	Practical assignment in the respective focus area<br><br>Individual site and time according to tandem – to be announced individually	Short greeting and flashlight on practical assignments (30 min.)<br>*Enke/Reisig/Idler*<br><br>From 10:00h: elaboration of project ideas in tandem in the ZeUS lecture rooms Großhadern (room assignment on site)<br><br>From 11:00h or 11:30h: personal talk with mentor (30 min., assignment on site); lunch break individually planned
**10:00**	Greeting and introduction<br>*Wildner/Idler*				
**10:30**	Health Promotion and Prevention: Fundamental Principles<br>*Heyn*				
11:15		Lunch break			
**12:00**	Lunch break	Project Planning (PH Action Cycle and Concrete Steps)<br>*Verdugo-Raab*			
12:45		Break			
**13:00**	Health Monitoring – Data Base for Prevention and Health Promotion<br>*Schulz*	Quality Development: The Good Practice Criteria in Prevention and Health Promotion<br>*Walentzak*			Short presentation by tandems of project ideas in plenary session, discussion<br>*Socher/Verdugo-Raab/Enke/Idler*
13:30	Break				
**13:45**	Public Communication of Prevention Messages<br>*Enke*	Break			
**14:00**		Practical Example: "Your Ticket to J1" (J1=adolescent health check)<br>*Bräuer/Hoffmann*			
14:45	Break				
**15:00**	Physician-Patient Communication<br>*Oberprieler*	End			End
16:00	End				

	Monday, September 21	Tuesday, September 22	Wednesday, September 23	Thursday, September 24	Friday, September 25 (Presentation and evaluation in room H5)
**08:00**			Students travel as a group to conference (at approx. 6:30h)	Registration and conference event attendance at students' discretion<br>Regensburg	Conference registration Regensburg<br><br>From 8:30h: Closing presentations<br>MeMPE Summer University Block I (mandatory attendance)<br>*Chair: Enke, Idler*
**9:00**			Registration and plenary address at the conference in Regensburg		
**10:00**	Project planning/elaboration in tandem (home office or room assignment by request, query by list and room assignment on September 18)<br><br>Talk with mentor (individual telephone consultation or appointment in person)	Project planning/elaboration in tandem (home office or room assignment by request, query by list and room assignment on September 18)			conference event attendance at students' discretion and individually planned lunch
**11:00**			Peer-to-peer presentation of projects (mandatory attendance)<br>*Idler*<br>Room ZH3		
**12:05**					Closing presentations MeMPE Summer University Block II (mandatory attendance)<br>*Chair: Enke, Idler*
13:00			conference event attendance at students' discretion		
**13:20**					Evaluation and closing remarks MeMPE Summer University (mandatory attendance)<br>*Idler*
**13:45**					If circumstances permit, participation in conference closing event
14:10					End and group return to Munich
16:45			End		
18:00	End	End			
18:15				End<br>*Optional: Café Picasso*	
