# Supplementary material for: Prevention and health promotion from theory to practice: The interprofessional MeMPE Summer University for students of Medicine, Master of Public Health and Epidemiology
Source: GMS J Med Educ. 2016 Nov 15;33(5):Doc72. doi: 10.3205/zma001071 (PMC5135415; doi:10.3205/zma001071)
Supplement: A3: Subscales of the pre and post questionnaires, with allocation of items, median values and range values [file JME-33-72-s-003.pdf]

A3: Subscales of the pre and post questionnaires, with allocation of items, median values and range values

**Pre questionnaire on self-evaluation**			
**Subscale**	**Item**	**Median**	**Range**
**Single item**	Item 1: My prior knowledge on the subjects of prevention and health promotion is extensive.	2.0	3.0
**Factual knowledge on the subjects of prevention and health promotion**	Item 2: I can name the contents of the Ottawa Charter for Health Promotion.	4.0	3.0
	Item 3: I can describe the Model of Salutogenesis.	2.0	4.0
	Item 4: I can name the fields of action and guiding principles of health promotion.	3.0	3,0
	Item 5: I can outline the (community-) setting approach.	3.0	3.0
	Item 6: I can discern target groups and points of time for intervention in the realm of prevention.	2.0	3.0
	Item 7: I can describe chains of events in prevention and desired outcomes in health promotion.	3.0	3.0
	Item 8: I can name the data sources of the Information System of Health Monitoring (Federal and Bavarian).	3.0	3.0
	Item 9: I can outline direct and indirect means of conveying public prevention messages.	3.0	3.0
	Item 10: I can describe different media uses as a basis for conveying public prevention messages.	3.0	3.0
	Item 11: I can name different forms of approach via mass communication and personnel communication.	3.0	3.0
	Item 12: I can name the fundamental principles of physician-patient communication.	2.0	4.0
	Item 13: I can name the core contents of communicating prevention information in physician-patient direct consultation.	2.0	3.0
	Item 14: I can describe strategies of communicating prevention information in physician-patient direct consultation.	3.0	3.0
	Item 15: I can name the steps in project planning.	3.0	4.0
	Item 16: I can name the criteria of Good Practice.	4.0	3.0
**Scientific work (application of factual knowledge, elaboration of the scientific project)**	Item 17: I can use the data sources of the Information System of Health Monitoring (Federal and Bavarian) for project work.	3.0	3.0
	Item 18: I can use the steps of project planning to design my own project.	3.0	3.0
	Item 19: I can use the criteria of Good Practice in designing my own project.	4.0	3.0
	Item 20: I am able to formulate a well-founded scientific problem statement.	3.0	3.0
	Item 21: I can elaborate a scientific project report according to specific guidelines.	2.0	3.0
	Item 22: I can elaborate a scientific project presentation according to specific guidelines.	2.0	2.0
	Item 23: My public presentation skills are good.	2.0	3.0
**Interprofessional work**	Item 24: I can elaborate a project together with a tandem partner.	2.0	3.0
	Item 25: I can conduct interprofessional exchange with a tandem partner.	2.0	2.0
	Item 26: I can conduct interprofessional exchange with peers (entire group of students).	2.0	2.0

Post questionnaire			
**Subscale**	**Item**	**Median**	**Range**
**Organization of the seminar and practical assignments**	Item 1: The events in the framework of the theoretical module were well-structured.	2.0	2.0
	Item 2: The events in the framework of the theoretical module were well-suited to each other.	2.0	3.0
	Item 3: The contents of the theoretical module and the practical module were well-suited to each other.	2.0	3.0
	Item 4: My practical assignment was well-organized by the medical practice/public health office/Helmholtz Zentrum.	1.0	3.0
	Item 5: The conference attendance event was well-organized by the project coordinators.	1.0	1.0
**Supervision during the seminar and practical assignment**	Item 6: My tandem's mentor took enough time for the supervision.	1.0	1.0
	Item 7: The supervision of my practical assignment (rural medical practice/public health office/Project Risikolotse) was good.	1.0	3.0
	Item 8: My supervisor provided a topic area for me in which I was able to develop a well-founded scientific problem statement.	2.0	4.0
**Didactics of the seminar and practical assignment**	Item 9: A pleasant working atmosphere prevailed during the seminar events.	1.0	1.0
	Item 10: I was given the opportunity to ask questions.	1.0	1.0
	Item 11: I was given the opportunity to collaborate actively.	2.0	3.0
	Item 12: The teaching content was illustrated using images, examples and cases.	2.0	3.0
	Item 13: I was encouraged to critically analyze the teaching content.	2.0	3.0
	Item 14: During my practical assignment (rural medical practice/public health office/Risikolotse), I became familiar with the practical implementation of prevention and health promotion.	2.0	3.0
	Item 15: If appropriate, please assess the practical assignment in a rural medical practice: I gained insight into the physician's function in a general practice in a rural setting.	1.0	3.0
	Item 16: If appropriate, please assess the practical assignment in a public health office: I was involved in the daily work within a prevention project.	3.0	3.0
	Item 17: If appropriate, please assess the practical assignment Risikolotse: I was involved in the project work in the form of workshops.	2.0	3.0
	Item 18: The exchange with my mentor helped me to develop a problem statement.	2.0	3.0
	Item 19: The interprofessional exchange with my peers (entire group of students) helped me to develop a problem statement.	2.0	3.0
**Single item**	Item 20: My knowledge on the subjects of prevention and health promotion is extensive.	2.0	2.0

Self-evaluation			
**Factual knowledge on the subjects of prevention and health promotion**	Item 21: I can name the contents of the Ottawa Charter for Health Promotion.	3.0	4.0
	Item 22: I can describe the Model of Salutogenesis.	2.0	3.0
	Item 23: I can name the fields of action and guiding principles of health promotion.	2.0	4.0
	Item 24: I can outline the (community-) setting approach.	2.0	3.0
	Item 25: I can discern target groups and points of time for intervention in the realm of prevention.	1.0	4.0
	Item 26: I can describe chains of events in prevention and desired outcomes in health promotion.	2.0	4.0
	Item 27: I can name the data sources of the Information System of Health Monitoring (Federal and Bavarian).	2.0	2.0
	Item 28: I can outline direct and indirect means of conveying public prevention messages.	2.0	4.0
	Item 29: I can describe different media uses as a basis for conveying public prevention messages.	2.0	2.0
	Item 30: I can name different forms of approach via mass communication and personnel communication.	1.0	2.0
	Item 31: I can name the fundamental principles of physician-patient communication.	2.0	3.0
	Item 32: I can name the core contents of communicating prevention information in physician-patient direct consultation.	2.0	3.0
	Item 33: I can describe strategies of communicating prevention information in physician-patient direct consultation.	2.0	3.0
	Item 34: I can name the steps in project planning.	1.0	1.0
	Item 35: I can name the criteria of Good Practice.	2.0	2.0
**Scientific work (application of factual knowledge, elaboration of the scientific project)**	Item 36: I can use the data sources of the Information System of Health Monitoring (Federal and Bavarian) for project work.	2.0	2.0
	Item 37: I can use the steps of project planning to design my own project.	1.0	1.0
	Item 38: I can use the criteria of Good Practice in designing my own project.	2.0	2.0
	Item 39: I am able to formulate a well-founded scientific problem statement.	2.0	2.0
	Item 40: I can elaborate a scientific project report according to specific guidelines.	1.0	2.0
	Item 41: I can elaborate a scientific project presentation according to specific guidelines.	1.0	2.0
	Item 42: My public presentation skills are good.	2.0	2.0
**Interprofessional work**	Item 43: I can elaborate a project together with a tandem partner.	1.0	2.0
	Item 44: I can conduct interprofessional exchange with a tandem partner.	1.0	2.0
	Item 45: I can conduct interprofessional exchange with peers (entire group of students).	1.0	2.0
**Overall assessment of learning results**	Item 46: The interprofessional exchange with my peers (entire group of students) during the Summer University contributed to a deeper understanding of the teaching contents.	2.0	4.0
	Item 47: The module topics were of interest to me.	2.0	2.0
	Item 48: The learning objectives became clear during the course of the Summer University.	2.0	4.0
	Item 49: I can give a summary of the content of the theoretical module.	2.0	2.0
	Item 50: Attending the theoretical module made preparation for the practical module easier.	2.0	4.0
	Item 51: I was able to appropriate content in the theoretical module that made me well-prepared for the	2.0	3.0

	formulation of a relevant scientific problem statement.		
	Item 52: I was able to formulate a well-founded scientific problem statement on the subject of prevention and health promotion in the framework of my project.	2.0	4.0
	Item 53: Presenting the project helped to improve my presentation skills.	2.0	3.0
	Item 54: Attending the Summer University enhanced my understanding of prevention and health promotion.	2.0	3.0
	Item 55: Overall, I learned a great deal by participating in the Summer University.	2.0	3.0
	Item 56: I find the topics of the Summer University relevant in regard to my future profession.	2.0	3.0
**Motivation for future participation in the Summer University**	Item 57: I attended the Summer University because…<br>A…the topic interested me.<br>B…the Summer University counts as credit in my compulsory curriculum.<br>C…friends attended the Summer University.<br>D…I wanted to enhance my knowledge on the subject of prevention and health promotion.<br>E…I wanted to familiarize myself with the rural medical profession.<br>F…I wanted to familiarize myself with professions in the public health office.<br>G…I wanted to familiarize myself with the operations of the prevention project “Risikolotse”.<br>H…other reasons.	not calculable; absolute frequency:<br>A: N=16;<br>B: N=7;<br>C: N=1;<br>D: N=8;<br>E: N=5;<br>F: N=10;<br>G: N=5;<br>H: N=2	not cal-culable

Median with decimal value (five-level Likert scale of 1.0=“fully agree” to 5.0=“fully disagree” regarding improvement in the corresponding subscale of self-evaluation).
